# Effects of flavonoids on sphingolipid turnover in the toxin-damaged liver and liver cells

**DOI:** 10.1186/1476-511X-7-1

**Published:** 2008-01-28

**Authors:** Nataliya A Babenko, Elena G Shakhova

**Affiliations:** 1 Department of Physiology of Ontogenesis, Institute of Biology, Kharkov Karazin National University, 4, Svobody pl., Kharkov, 61077, Ukraine

## Abstract

**Background:**

The ceramide generation is an early event in the apoptotic response to numerous stimuli including the oxidative stress and ceramide analogs mimic the stress effect and induce apoptosis. Flavonoids of German chamomile are reported to exhibit the hepatoprotective effect. Flavonoids affect sphingolipid metabolism and reduce the elevated ceramide level in the aged liver. In the present paper, the ceramide content and production in the CCl_4_- and ethanol-treated liver and hepatocytes as well as the correction of sphingolipid metabolism in the damaged liver using the mixture of German chamomile flavonoids (chamiloflan) or apigenin-7-glucoside (AP7Glu) have been investigated.

**Results:**

The experiments were performed in either the rat liver or hepatocytes of normal, CCl_4_- and ethanol-treated or flavonoid- and toxin plus flavonoid-treated animals. [^14^C]palmitic acid and [methyl-^14^C-phosphorylcholine]sphingomyelin were used to investigate the sphingolipid turnover. Addition of the CCl_4 _or ethanol to isolated hepatocyte suspensions caused loss of cell viability and increased the lactate dehydrogenase release from the cells into supernatant and ceramide level in the cells. CCl_4 _administration to the rats enlarged ceramide mass as well as neutral sphingomyelinase (SMase) activity and reduced ceramide degradation by the neutral ceramidase. Pretreatment of isolated hepatocytes with flavonoids abrogated the CCl_4 _effects on the cell membrane integrity and normalized the ceramide content. Flavonoid administration to the rats normalized the elevated ceramide content in the damaged liver via neutral SMase inhibition and ceramidase activation.

**Conclusion:**

The data obtained have demonstrated that flavonoids affect sphingolipid metabolism in the CCl_4_- and ethanol-damaged liver and liver cells. Flavonoids normalized activities of key enzymes of sphingolipid turnover (neutral SMase and ceramidase) and ceramide contents in the damaged liver and liver cells, and stabilized the hepatocyte membranes.

## Background

Sphingolipids are a structurally diverse group of compounds composed of a long-chain sphingoid base backbone and amino group, which is often substituted with a long-chain fatty acid. They are found primarily in cell membranes and are known to play roles in cell-cell and cell-matrix interactions, and as second messengers [1-4]. In the last years, the regulatory role of ceramide generated by the sphingomyelin (SM) cycle has received increasing attention. Sphingomyelinase (SMase) and SM synthase are the key enzymes of the SM cycle. SMase removes phosphocholine from SM to produce ceramide. Once generated, ceramide could be converted to SM by the SM synthase activation. In intact cells, the rapid ceramide generation is an early event in the apoptotic response to numerous stimuli including the oxidative stress and the ceramide analogs mimic the stress effect and induce apoptosis [2]. It has been recently demonstrated that acid SMase plays a significant role in hepatocellular apoptosis and liver damages induced by TNF-α [5]. However, hepatoblastoma cell Hep G2 treatment by ethanol was accompanied by the neutral SMase activation, ceramide accumulation and the significant increase of caspase-3 activity and apoptotic cell death [6]. Although the ceramides are well known to cause cell death by inducing apoptosis, they can also cause cell death by necrosis. It has been determined that incubation of the suspension of freshly isolated rat hepatocytes in the presence of exogenous short-chain ceramides (C_2_, C_6_, C_8_) induced hepatocellular death by necrosis and not apoptosis as confirmed by the morphology and the absence of internucleosomal DNA cleavage [7]. The ceramide induced hepatocyte necrosis was associated with adenosine triphosphate depletion and mitochondrial depolarization suggesting that the ceramides caused mitochondrial dysfunction.

A strong correlation exists between the changes in the SMase activity and in the level of oxidation products caused by either reduced or oxidized glutathione in the liver cells [8] and cells of other types [9,10]. Generation of ceramide has been attributed to serum starvation, Fas-induced apoptosis [1-4], and cellular senescence studies [11]. The fact that glutathione depletions frequently observed in most of these situations suggests that the levels of cellular glutathione regulates the ceramide generation. It has been determined that at physiologically relevant concentrations glutathione inhibits the neutral magnesium-dependent SMase and that the depletion of cellular glutathione results in the hydrolysis of SM and generation of ceramide [10]. Studies with analogs and fragments of glutathione demonstrated that the structural requirements for inhibition reside in the γ-glutamyl-cysteine moiety of glutathione. This structural specificity in inhibiting neutral SMase suggests that glutathione may function as a specific allosteric regulator of enzyme.

It has been determined that the flavonoids of German chamomile (chamiloflan) normalized sphingolipid metabolism in the liver of old rats [11] with an extremely low, as compared to young animals, level of hepatocellular glutathione [12]. Both the long- and the short-term effects of chamiloflan on sphingolipid turnover have been determined. The flavonoids reducing the SMase activity decrease the elevated ceramide mass in the old liver down to the level of adult rats. It has been supposed that, the flavonoids of chamiloflan could reduce the neutral SMase activity via the elevation of glutathione in the liver.

Such flavonoids, as apigenin-7-glucoside (AP7Glu), luteolin-7-glucoside (LU7Glu) and quercitin prevented the glutathione depletion and lipid peroxidation induced by an acute intoxication with carbon tetrachloride (CCl_4_), ethanol, acetominophen and bromobenzene in the liver and in the rats with biliary obstruction [13-16]. The best-described property of almost every group of flavonoids is their capacity to act as antioxidants [17]. The flavones and flavonols (apigenin, luteolin, quercetin, rutin and others) seem to be the most powerful flavonoids for protecting a body against reactive oxygen species. These compounds have the potential to scavenge and quench various radicals (oxygen-centered, carbon-centered, alkoxyl, peroxyl, or phenoxyl radicals) and reactive oxygen species. The flavonoids may have an additive effect to the endogenous scavenging compounds and also increase the function of the endogenous antioxidants. The flavonoids interrupt the lipid peroxidation chain reaction and thereby prevent glutathione depletion that plays a critical role in cellular defense against oxidative stress.

The present paper considers the influence of plant polyphenols on the CCl_4_- or ethanol-treated liver cell injury and sphingolipid turnover. Significant increase in the levels of ceramide, ceramide/SM ratio and neutral SMase activity has been observed in the cells of CCl_4_- and ethanol-treated liver. The flavonoids restored the ceramide level and decreased the elevated ceramide/SM ratio in the damaged liver of CCl_4_-treated animals or isolated hepatocytes. The intracellular accumulation of ceramides in the toxin-treated cells and liver could be prevented by using the flavonoids of chamiloflan via a mechanism involving an inhibition of the neutral SMase and the ceramidase activation.

## Results and Discussion

**Figure 1:**
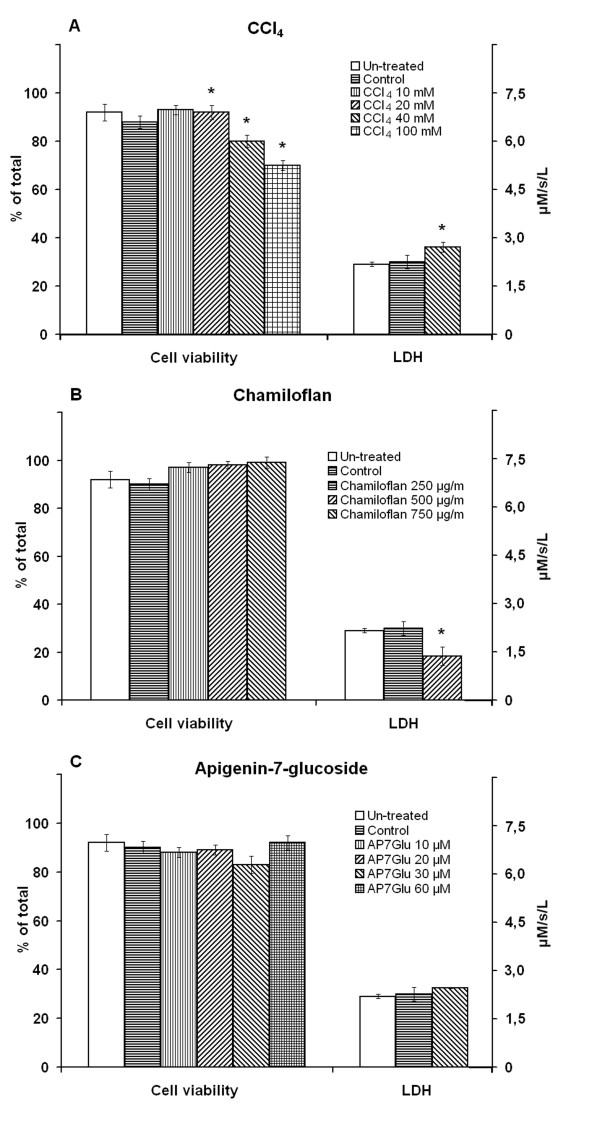
**Effects of the CCl_4 _and flavonoids on the hepatocyte death**. The cells (10^6^/ml) were incubated at 37°C in Eagle medium containing 10–100 mM CCl_4 _for 2 h. Control cells were incubated in the presence of 30 mM ethanol for 2 h. Hepatocytes were incubated in the presence of the 250–750 μg/ml chamiloflan, or 10–60 μM AP7Glu for 4 h. The time of the control cell suspension incubation (in the presence of 30 mM ethanol) was 4 h. To determine the cells viability, the trypan blue exclusion into hepatocytes and LDH release into supernatant have been studied using commercially available kits. Results are mean ± S.E. of six experiments performed in duplicate. * P < 0.05, CCl_4 _-treated vs. control.

**Figure 2:**
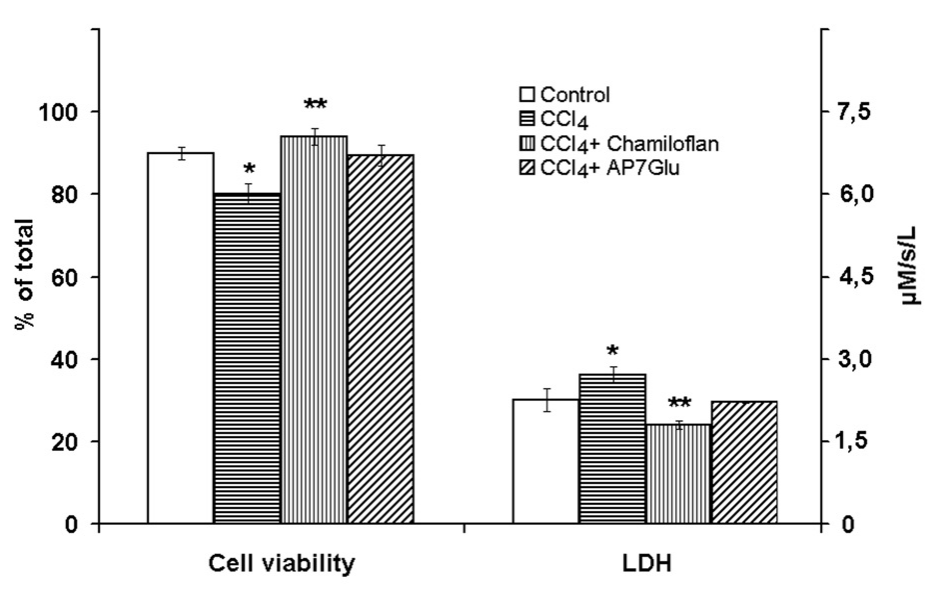
**Effects of the flavonoids on the hepatocyte death induced by the CCl_4_**. The cells (10^6^/ml) were pre-incubated at 37°C in Eagle medium for 2 h followed by the 40 mM CCl_4 _addition and suspension was incubated for 2 h. In some cases chamiloflan (500 μg/ml) or AP7Glu (30 μM) were added to the culture medium 2 h prior the CCl_4_addition and cells incubated for 4 h. Control cells were incubated in the presence of 30 mM ethanol for 4 h. To determine the cells viability, the trypan blue exclusion into hepatocytes and LDH release into supernatant have been studied using commercially available kits. Results are mean ± S.E. of six experiments performed in duplicate. * P < 0.05, CCl_4_-treated vs. control; ** P < 0.05, CCl_4_+flavonoid-treated vs. CCl_4_-treated.

Addition of the CCl_4 _to isolated hepatocyte suspensions caused loss of cell viability and an increased LDH release from the cells into supernatant in a concentration dependent manner (Figure 1A). Trypan blue staining and the LDH measurements in the culture medium indicated that there was no disruption of the normal toxin-untreated hepatocyte membranes with any of the chamiloflan flavonoids tested (Figure 1B, C). Chamiloflan or AP7Glu pretreatment of hepatocytes prevented the CCl_4_-induced cell damage. As shown in Figure 2, chamiloflan and AP7Glu treatments resulted in initial drop of trypan blue inclusion into the cells and the LDH release from the hepatocytes into culture medium of the CCl_4_-treated cells.

**Figure 3:**
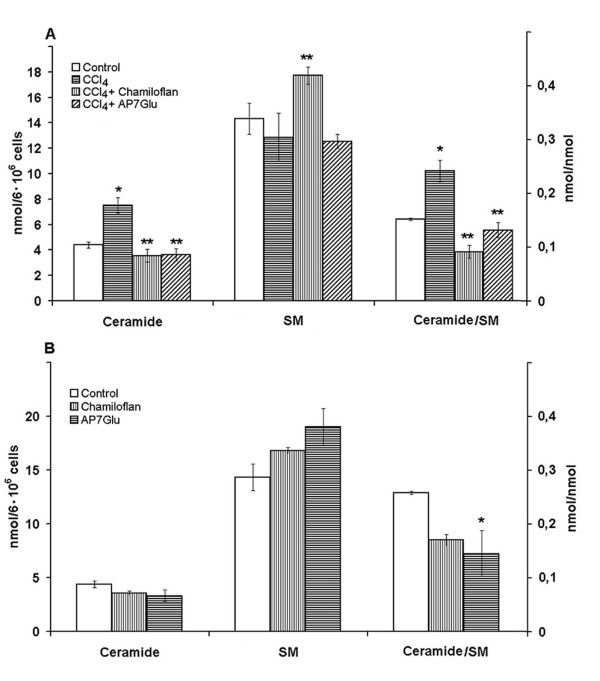
**Effects of the flavonoids on ceramide and sphingomyeline contents in the normal and CCl_4_-treated hepatocytes**. The cells (10^6^/ml) were pre-incubated at 37°C in Eagle medium for 2 h followed by the 40 mM CCl_4 _addition and suspension was incubated for 2 h. In some cases the chamiloflan (500 μg/ml) or AP7Glu (30 μM) were added to the culture medium 2 h prior the CCl_4 _addition and the cells incubated for 4 h. Control cells were incubated in the presence of 30 mM ethanol for 4 h. Lipid contents in the cells were determined as described in the "Materials and Methods". Results are mean ± S.E. of six experiments performed in duplicate. * P < 0.05, CCl_4_-treated vs. control; ** P < 0.05, CCl_4_+flavonoid-treated vs. CCl_4_-treated.

**Figure 4:**
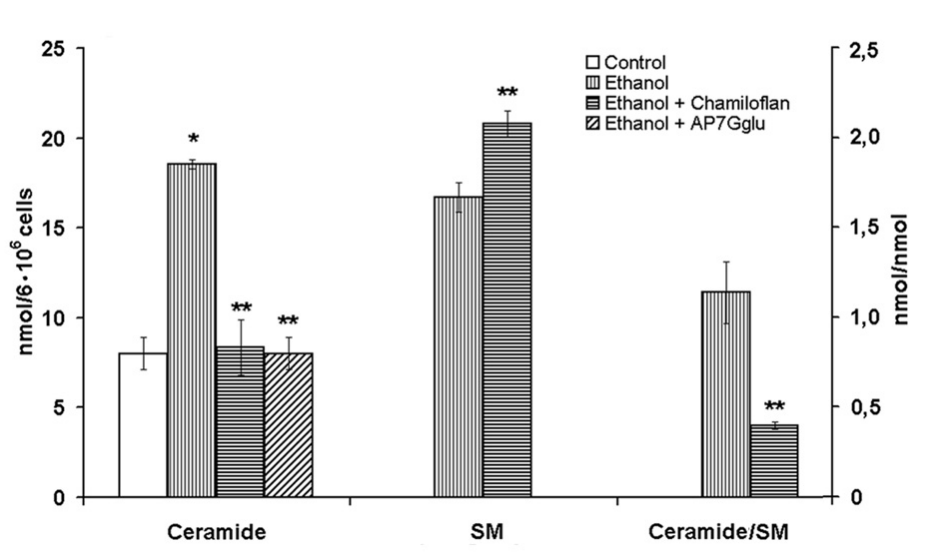
**Effects of the flavonoids on ceramide and sphingomyeline contents in the normal and ethanol-treated hepatocytes**. The cells (10^6^/ml) were incubated at 37°C in Eagle medium containing 70 mM ethanol for 4 h. Control cells were incubated without any additions for 4 h. In some cases, the chamiloflan (500 μg/ml) or AP7Glu (30 μM) were added to the culture medium together with the ethanol addition and cells incubated for 4 h. The ethanol concentration in the flavonoid-treated cell suspension was 70 mM. Lipid contents in the cells were determined as described in the "Materials and Methods". Results are mean ± S.E. of six experiments performed in duplicate. * P < 0.05, ethanol-treated vs. control; ** P < 0.05, ethanol+flavonoid-treated vs. ethanol-treated.

It is known, that the synthesis and the degradation of sphingolipids are regulated by oxidative stress [18]. The oxidative stress is often associated with the endogenous ceramide accumulation in the injured cells. The glutathione has been found to be a powerful negative regulator of the neutral SMase activity in the different cells [9,10]. The elevated content of the ceramide and ceramide/SM ratio has been found in the CCl_4_- (Figure 3A) or ethanol-treated (Figure 4) hepatocytes and in the liver of CCl_4_-treated rats (Table 3). It is well documented that cell death induced by ethanol is associated with stimulation of neutral and acidic SMases and ceramide generation, as well as with the activation of the stress-related kinases, c-Jun N-terminal kinase, p38 mitogen-activated protein kinase and extracellular signal-regulated kinase (ERK) pathways [6,19,20]. It has been determined, that pharmacological inhibitors of these kinases largely prevent the apoptosis induced by the ethanol and C_2_-ceramide [19]. These results strongly suggest that ethanol is able to stimulate the SMase-ceramide pathway, leading to the activation of signaling pathways implicated in the cell death.

**Table 3:** Effects of the chamiloflan on the ceramide content in the liver of the normal and CCl_4_-treated rats.

Animals	Ceramide	Ceramide/SM
Un-treated	6.74 ± 0.54	0.131 ± 0.010
Control (1)	6.95 ± 0.45	0.121 ± 0.015
Chamiloflan-treated	6.83 ± 0.23	0.112 ± 0.005
Control (2)	5.67 ± 0.46	0.117 ± 0.010
CCl_4_-treated	7.34 ± 0.47 *	0.162 ± 0.013 *
Chamiloflan + CCl_4_-treated	5.93 ± 0.51	0.135 ± 0.017

The rats were fed by the chamiloflan (160 mg/kg body weight) or ethanol (1 %, 5 ml/kg body weight) (control 1 group) intragastrically daily for 6 days. The CCl_4 _(50 % in corn oil, 4 ml/kg body weight) was injected subcutaneously daily for 4 days. Some of the CCl_4_- treated rats were fed by the chamiloflan (160 mg/kg body weight) daily for 6 days intragastrically. Control (2) rats were injected subcutaneously by the corn oil (2 ml/kg body weight) daily for 4 days and fed by the ethanol (1 %, 5 ml/kg body weight) daily for 6 days. The animals were starved overnight prior the experiments. The amount of the ceramides in the liver homogenate was represented as nmol per mg of protein. Results are mean ± SE of 10 experiments performed in duplicate. * R < 0.05, CCl_4_-treated rats vs. control.

**Figure 5:**
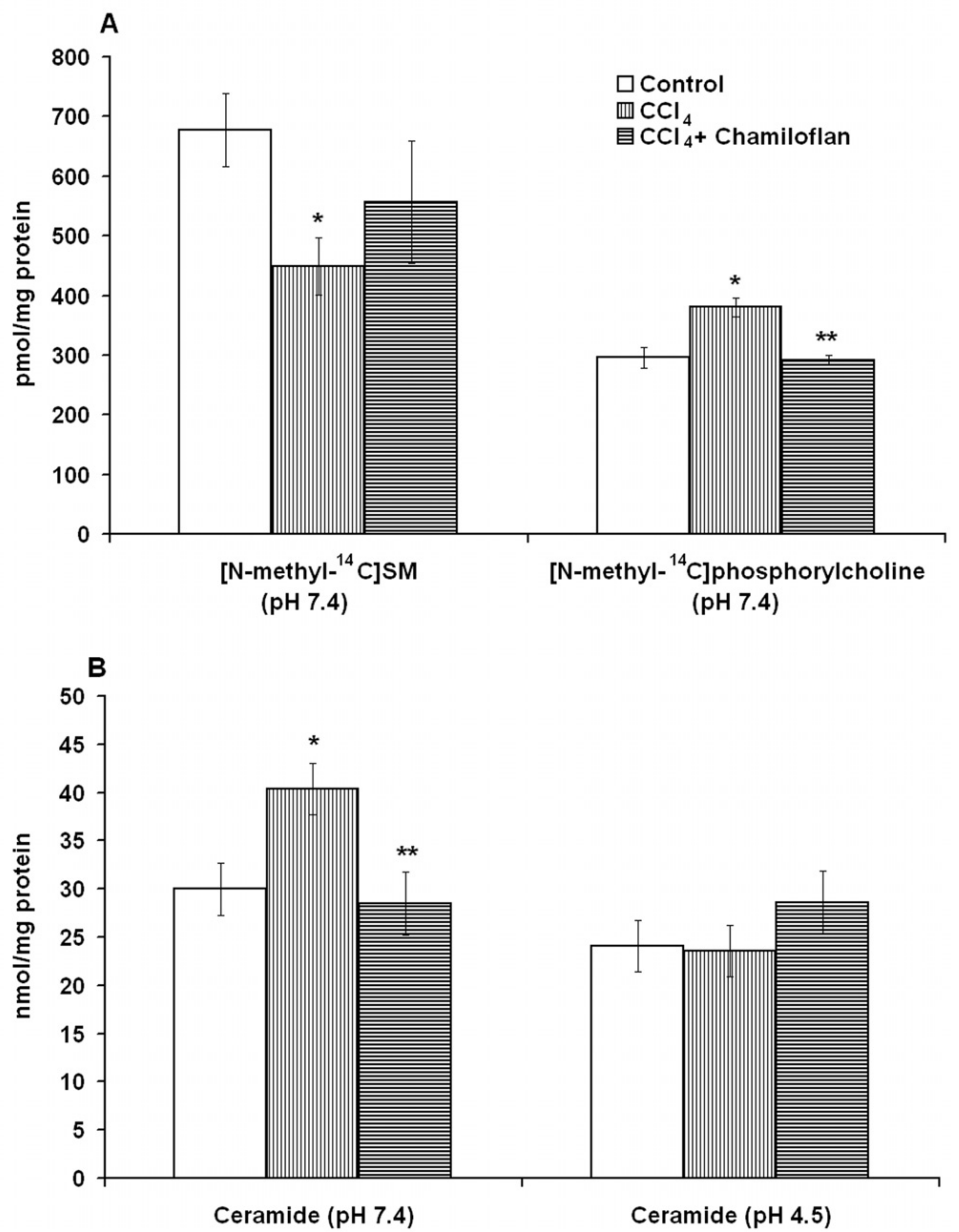
**Effects of the flavonoids on the shingomyelinase activity and ceramide production induced in the liver by the CCl_4_**. CCl_4 _(50% in corn oil, 4 mg/kg body weight) was injected to rats subcutaneously daily for 4 days and fed by ethanol (1 %, 5 ml/kg body weight) daily for 6 days Some of the animals were fed by chamiloflan (160 mg/kg body weight) daily for 4 days together with CCl_4 _injections and 2 days after last CCl_4 _treatment. Control rats were injected subcutaneously by corn oil (2 ml/kg body weight) daily for 4 days and fed by ethanol (1 %, 5 ml/kg body weight) daily for 6 days. Liver homogenates were used for the determination of the neutral SMase activity (pH 7.4) (A) and ceramide generation under neutral (pH 7.4) and acidic (pH 4.5) conditions (B) as described in the "Materials and Methods". The [methyl-^14^C-phosphorylcholine]sphingomyelin as a substrate was used. Results are mean ± S.E. of six experiments performed in duplicate. * P < 0.05, CCl_4_-treated rats vs. control; ** P < 0.05, CCl_4_+flavonoid-treated rats vs. CCl_4_-treated.

It has been demonstrated that CCl_4 _as well as ethanol lead to the pronounced accumulation of ceramide in the liver cells and stimulate the sphingolipid turnover in the liver. Using the [methyl-^14^C-phosphorylcholine]SM, it has been determined that under the neutral pH conditions the [^14^C-methyl]SM content drop, while [^14^C-methyl]phosphorylcholine release from the [^14^C-methyl]SM increased in the liver homogenates of CCl_4_-treated rats, as compared to the control animals (Figure 5A). The [^14^C-methyl]SM hydrolyses at neutral pH was accompanied by ceramide production in the liver homogenates of the toxin-treated animals as compared with the control rats (Figure 5B). The administration of the CCl_4 _to the rats did not change the ceramide production (Figure 5B) and [^14^C-methyl]SM degradation in the liver homogenates under the acidic pH (data not shown). The results obtained strongly demonstrated that the acidic SMase was not implicated in ceramide accumulation in the CCl_4_-treated liver.

**Figure 6:**
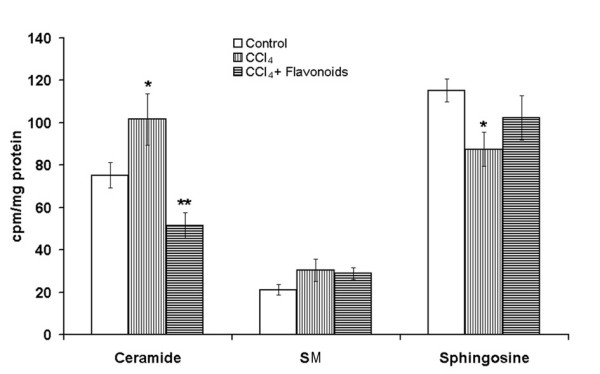
**Effects of the flavonoids on the ceramide turnover induced in the liver by the CCl_4_**. CCl_4 _(50% in corn oil, 4 mg/kg body weight) was injected to rats subcutaneously daily for 4 days and fed by ethanol (1 %, 5 ml/kg body weight) daily for 6 days Some of the animals were fed by chamiloflan (160 mg/kg body weight) daily for 4 days together with CCl_4 _injections and 2 days after last CCl_4_treatment. Control rats were injected subcutaneously by corn oil (2 ml/kg body weight) daily for 4 days and fed by ethanol (1 %, 5 ml/kg body weight) daily for 6 days. Liver homogenates were used for the determination of ceramide turnover. To study the ceramide conversion to SM and sphingosyne, the [^14^Cpalmitate-pre-labeled [^14^C]ceramide was used. Lipid extraction and separation were determined as described in the "Materials and Methods". Results are mean ± S.E. of six experiments performed in duplicate. * P < 0.05, CCl_4_-treated vs. control; ** P < 0.05, CCl_4_+flavonoid-treated vs. CCl_4_-treated.

Once generated, ceramide can accumulate in the cell or may be converted into a variety of metabolites. The deacylation of ceramide by either a neutral or acid ceramidase yields sphingosine, which may then be phosphorylated by sphingosine kinases to sphingosine 1-phosphate. Ceramide may also be converted into SM by a transfer of phosphorylcholine from phosphatidylcholine (PC) to ceramide via SM syntase. [21,22]. It was determined that the modulation of SM synthase and ceramidase activities were accompanied by the changes of ceramide levels in the cells [23,24]. To study the impact of SM synthase and ceramidase in ceramide accumulation in the toxin-treated liver the [^14^C]palmitate-prelabeled ceramide turnover was analyzed. The administration of the CCl_4 _to the rats led to the reduction of the neutral ceramidase activity, the [^14^C]sphingosine production and the elevation of the [^14^C]palmitate-prelabeled ceramide level in the liver homogenates (Figure 6). However, CCl_4 _had no effect on the [^14^C]ceramide conversion to SM. The obtained results demonstrated that the decrease of the ceramide turnover via ceramidase could led to the lipid accumulation in the toxin-treated liver.

**Figure 7:**
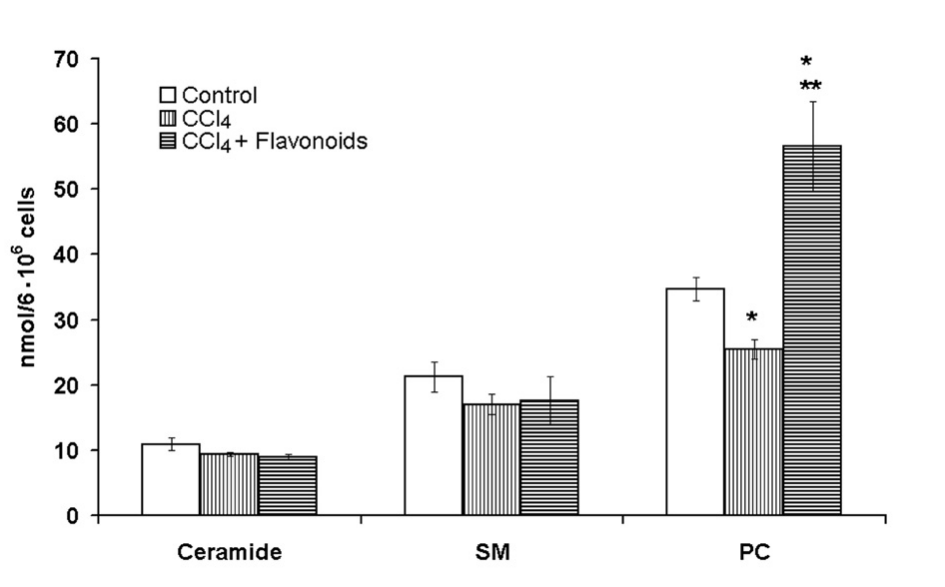
**Effects of the flavonoids on ceramide, sphingomyelin and phosphatidylcholine release from the hepatocytes pre-treated by the CCl_4_**. The cells (10^6^/ml) were incubated at 37°C in Eagle medium containing 30 μM ethanol for 2 h followed by 40 mM CCl_4_treatment for 2 h. Chamiloflan (500 μg/ml) was added to the culture medium 2 h prior 40 mM CCl_4 _addition and cells incubated for 4 h. Control cells were incubated in the media containing 30 μM ethanol for 4 h. Lipid contents in the culture medium were determined as described in the "Materials and Methods". Results are mean ± S.E. of six experiments performed in duplicate. * P < 0.05, CCl_4_-treated vs. control; ** P < 0.05, CCl_4_+flavonoid-treated vs. CCl_4_-treated.

Recent studies have indicated that ceramide generated in the liver is secreted into the bloodstream in the form of very-low-density lipoproteins (VLDL) and low-density lipoproteins (LDL) [25,26]. Importantly, the changes in the hepatic serine-palmitoyl transferase activity (the rate-limiting step in the *de novo *ceramide synthesis) affect the rate of the ceramide secretion. The activation of the enzyme by the addition of palmitic acid leads to an elevation in VLDL and LDL ceramide. And vice versa, the inhibition of the *de novo *ceramide synthesis by Fumonisin B1 prevents the incorporation of ceramide in VLDL and LDL. It has been determined, that CCl_4 _decreases the lipoprotein synthesis in the liver cells and its transport into the bloodstream [27]. The release of the PC from the CCl_4_-treated hepatocytes was less in the magnitude then that from the control cells (Figure 7). However, CCl_4 _had no effect on the ceramide and SM release into the culture media as compared to the control cells. This result eliminates the role of the ceramide and SM transport from the cells in the CCl_4_-induced sphingolipid accumulation in the isolated hepatocytes.

The flavonoids are reported to exhibit a wide variety of biological effects, including antioxidant and free radical scavenging activities, as well as hepatoprotective and anti-inflammatory effects [17]. Because of their antioxidant properties, the flavonoids were able to reduce the damage of hepatocytes and liver induced by chemicals. The intragastric administration of the mixture of chamomile flavonoid isomers (such as apigenin, luteolin, AP7Glu, LU7Glu, isorhamnetin and quercetin (chamiloflan)) to the adult rats nullified the CCl_4_-induced increase of serum alanine aminotransferase, aspartate aminotransferase, and gamma-glutamyl transpeptidase activities in blood serum and prevented the hepatocellular fatty degeneration [28]. The intracellular accumulation of ceramides during aging can be prevented by using the flavonoids of chamiloflan via a mechanism that involves an inhibition of the acid and neutral SMases [11].

The pre-treatment of hepatocytes with flavonoids of chamiloflan or AP7Glu nullified the CCl_4_- (Figure 3A) or ethanol-induced (Figure 4) increase of ceramide and ceramide/SM ratio. Chamiloflan increased the SM mass in the toxin treated cells (Figure 3A). However, AP7Glu did not change the phospholipid content in the CCl_4_-treated hepatocytes. The administration of the flavonoids to the CCl_4_-treated rats reduced enlarged ceramide mass and ceramide/SM ratio in the liver, as compared to the control animals (Table 3). While the flavonoids that were used had no effect on the untreated normal isolated hepatocytes (Figure 3B) and rat liver (Table 3).

The administration of the flavonoids to the CCl_4_-treated rats reduced the neutral SMase activity measured by the release of the [^14^C]phosphorylcholine from the [^14^C-methyl]SM (Figure 5A) and ceramide production in the liver homogenates (Figure 5B). However, the flavonoids did not change the [^14^C-methyl]SM degradation (data not shown) and the ceramide production under acid pH in the liver of the CCl_4_-treated rats (Figure 5B). The results that were obtained demonstrated that the neutral SMase could be involved in the flavonoid-associated normalization of ceramide production in the toxin-damaged liver cells. Besides, other pathways of the ceramide turnover could be influenced by the flavonoids of chamomile. The administration of the flavonoid to the CCl_4_-treated rats decreased the level of the [^14^C]palmitate-pre-labeled ceramide in the liver homogenates and increased its degradation by the neutral ceramidase to the [^14^C]sphingosine (Figure 6). However, chamiloflan did not stimulate the [^14^C]palmitate-pre-labeled ceramide conversion to the SM (Figure 6) and ceramide transport from the CCl_4_-treated hepatocytes to the culture media (Figure 7). At the same time, the flavonoids increased the PC release from the toxin-damaged hepatocytes into culture media (Figure 7) and enhanced the lipoprotein synthesis and transport from the liver cells [17].

The mechanism by which the flavonoids could affect the sphingolipid turnover has not been explored. It is known that the generation of ceramide can be regulated by numerous factors. From the present study, two factors are worthy of discussion. First, it is known, that AP7Glu, LU7Glu and quercitin prevented the glutathione depletion and lipid peroxidation induced by an acute intoxication with the CCl_4 _or ethanol in the liver [13,14]. The flavonoids of chamomile (chamiloflan) normalized the ceramide levels soon after flavonoids addition to the toxin-treated hepatocytes or injection to the rats. The glutathione has been found to be a powerful negative regulator of neutral SMase activity in the different cells [9,10]. Thus, the flavonoids of chamiloflan could reduce the neutral SMase activity via the elevation of glutathione in the liver of the CCl_4_- or ethanol-treated rats or the isolated liver cells. Second, it is well known that the CCl_4_- induced production of the reactive oxygen species coincides with the phospholipase A_2 _(PLA_2_) activation and arachidonic acid (AA) release in the damaged liver cells [29]. AA is a known activator of SMase [30] and inhibitor of the ceramidase activities in the different cells [31]. It has been determined that the flavonoids, especially the apeginine and gurcetine are the inhibitors of PLA_2 _activity in the toxin-damaged cells [32-34]. To summarize, the flavonoid-dependent PLA_2 _inhibition may be responsible for the decreased neutral SMase and the increased ceramidase activities in the CCl_4_-treated liver cells. Further investigations are needed to elucidate these conclusions.

## Conclusion

Our study suggests that the CCl_4 _activates the sphingolipid turnover in the hepatocytes and liver and these effects mimic the ethanol action on the liver cells. The flavonoids of chamomile affect the SM and ceramide metabolism in the toxin-damaged liver and cultured hepatocytes. Both the *ex vivo *and the *in vivo *effects of the plant flavonoids have been determined. The addition of flavonoid to the culture media or administration to the rats normalized the elevated ceramide content in the damaged hepatocytes or the liver possibly via the neutral SMase inhibition and the ceramidase activation. The flavonoid-induced alterations in sphingolipid turnover coincided with the stabilization of the toxin-treated hepatocyte membranes and prevented the cell death.

## Materials and methods

### Materials

[^14^C]palmitic acid (56.0 mCi/mmol, Amersham, GE Healthcare UK), [methyl-^14^C-phosphorylcholine]sphingomyelin (58 mCi/mmol) – Amersham Corp., Silica Gel Woelm TLC without any binder (ICN Pharmaceuticals GmbH & Ca.). Lipid standards (ceramide, sphingosine, PC, SM) were obtained from Sigma (USA). Flavonoids of German chamomile (chamiloflan), AP7Glu and LU7Glu were produced by the State Scientific Center of Drugs (Kharkov, Ukraine). Chamiloflan contains the flavones (apigenin, luteolin, AP7Glu and LU7Glu) and flavonols (isorhamnetin and quercetin). Lactate dehydrogenase (LDH UV SCE) kit was from Felecit Diagnostic, Dnepropetrovsk, Ukraine. Other chemicals used were of chemically pure grade.

### Animals

The 90-day-old male Wistar rats were used in the experiments. They had a free access to a standard diet and water *ad libitum *and were kept at 24°C on a cycle of 12 h light/12 h darkness. The animals were divided into several groups (Table 1). The rats were fed by chamiloflan or ethanol intragastrically. CCl_4 _was injected subcutaneously. The animals were starved overnight prior to the experiment. All rats were anaesthetized with ketamine (75 mg/kg) and sacrificed by decapitation. The livers were obtained 24 h after the last feeding of the rats by chamiloflan or ethanol or after CCl_4 _administration

**Table 1:** Experimental protocol: experiments with the animals.

Animals	Treatments:			
	
	Ethanol (1 %)	Corn oil	CCl_4 _(50 %, dissolved in corn oil)	Chamiloflan (dissolved in 1 % ethanol)
Un-treated	-	-	-	-
Control (1)	5 ml/kg body weight for 6 days	-	-	-
Chamiloflan-treated	-	-	-	160 mg/kg body weight for 6 days
Control (2)	5 ml/kg body weight for 6 days	2 ml/kg body weight for 4 days	-	-
CCl_4_-treated	5 ml/kg body weight for 6 days	-	4 ml/kg body weight for 4 days	-
Chamiloflan + CCl_4_-treated	-	-	4 ml/kg body weight for 4 days	160 mg/kg body weight for 6 days

### Experiments with Liver Homogenates

The liver was perfused with 0.9% NaCl, then removed and washed in Krebs-Henseleit buffer, pH 7.4, containing 2 mM CaCl_2 _and 0.2% BSA. Liver homogenates prepared in 50 mM Tris-HCl, pH 7.4, 1 mM EDTA, 10 mM magnesium chloride, 0.65% Triton X-100 or 50 mM sodium acetate, pH 5.0 were used to determine the SMases activities as described below. For lipid separation the liver homogenates were prepared in the Krebs-Henseleit buffer, pH 7.4. The lipids were extracted and analyzed as described below.

### Experiments with hepatocytes

Hepatocytes were isolated from the 90-day-old male Wistar normal rats by the method described in [35]. The preparation of hepatocytes started between 9:00 and 10:00 a.m. The cells (10^6^/ml) were cultured on the 60 mm Petri dishes coated with rat tail collagen [36] in Eagle medium containing 10% fetal calf serum, 100 units/liter streptomycin, 100 units/liter penicillin, 13 mg/ml gentamycin in a humidified atmosphere of 5% CO_2 _in a tissue culture incubator at 37°C. The rat hepatocytes were incubated in the presence of 250–750 μg/ml chamiloflan or 10–60 μM AP7Glu for 4 h. The control cell cultures contained 30 μM ethanol. In some cases the hepatocytes were incubated in the presence of 10–100 mM CCl_4 _or 70 μM ethanol. The treatment protocol was demonstrated in Table 2. The cells were released from the dishes by a mild trypsin treatment. The hepatocytes were allowed to settle and the pellet was resuspended in the Krebs-Henseleit buffer, pH 7.4, containing 2 mM CaCl_2_, 25 mM HEPES, 0.1% BSA. To determine the cells viability, the trypan blue exclusion into hepatocytes and LDH release into supernatant have been studied using commercially available kits. To obtain the [^14^C]-labeled ceramide the isolated hepatocytes were incubated in the Eagle medium containing 10% fetal calf serum, 100 units/liter streptomycin, 100 units/liter penicillin, 13 mg/ml gentamycin and 2.5 μCi/ml [^14^C]palmitic acid for 24 h. During the incubation, the cells were maintained at 37°C and humidified atmosphere of 5% CO_2 _in a tissue culture incubator. Before the lipid extraction, the cells were washed twice with the Krebs-Henseleit buffer, pH 7.4, containing 2 mM CaCl_2_, 25 mM HEPES, 0.1% BSA. The lipids were extracted and [^14^C]ceramide was isolated as described below.

**Table 2:** Experimental protocol: experiments with the hepatocytes.

Cells**	Treatments*:			
	
	Ethanol	Chamiloflan	AP7Glu	CCl_4_
Un-treated	-	-	-	-
Control (for CCl_4_-treated)	+	-	-	-
CCl_4_-treated	-	-	-	+
Control (for flavonoid- treated)	+	-	-	-
Chamiloflan-treated	-	+	-	-
AP7Glu-treated	-	-	+	-
Chamiloflan + CCl_4_-treated	-	+	-	+
AP7Glu + CCl_4_-treated	-	-	+	+
Control (for ethanol-treated)	-	-	-	-
Ethanol-treated	+	-	-	-
Chamiloflan + ethanol-treated	+	+	-	-
AP7Glu + ethanol-treated	+	-	+	-

*- The flavonoids and CCl_4 _were dissolved in the ethanol. The time of incubation with the flavonoids: 4 h and with the CCl_4_: 2 h. **- Control cell media for the CCl_4_- and flavonoids-treated hepatocytes contained 30 mM ethanol. Control cell media (for 70 mM ethanol-treated cells) had no additions. Ethanol-treated cell media contained 70 mM ethanol.

### Determination of sphingolipid metabolism

The activities of neutral and acid SMases were determined using the liver cells homogenates and [methyl-^14^C-phosphorylcholine]sphingomyelin (58 mCi/mmol) [37]. The reaction mixture contained liver homogenate in 50 mM Tris-HCl, pH 7.4, 1 mM EDTA, 10 mM magnesium chloride, 0.65% Triton X-100 and 2 mM [methyl-^14^C]sphingomyelin in a final volume of 200 μl. The reaction proceeded up to 1 h at 37°C and was then terminated by the addition of 1.5 ml of chloroform/methanol (1:2, v/v) followed by 1 ml of chloroform and 1 ml of H_2_O. The mixture was centrifuged for 5 min at 3,000 rpm. After phase separation, a portion of the upper, aqueous phase containing [^14^C]phosphorylcholine was removed and the radioactivity determined by liquid scintillation counting. To determine the remained [methyl-^14^C]sphingomyelin and unlabeled ceramide formation the lower phase was analyzed as described below. To study the ceramide conversion to SM and sphingosyne, the [^14^Cpalmitate-pre-labeled [^14^C]ceramide was used [38]

### Extraction and Separation of Lipids

The lipids were extracted according to the Bligh and Dyer protocol [39]. The chloroform phase was collected and dried under N_2 _at 37°C. The lipids were redissolved in chloroform/methanol (1:2, v/v) and applied on the TLC plates. The plates for lipid separation were prepared from Silica Gel Woelm TLC without any binder. The layer thickness was 0.25 mm. For the PC separation the solvent system: chloroform/methanol/acetic acid/water (25:15:4:2, v/v) was used. For the ceramide and SM separation, the plates with loaded lipid extract were developed with diethyl ether. When the solvent front reached the top of the plate, the plate was removed from the chamber, dried, and developed again with chloroform/methanol/water (40:10:1, v/v). In this case, the solvent front was allowed to reach 2/3 of the plate length. The regions migrating with the standart ceramide and SM were scraped from the plate and the ceramide eluted from the silica with 1 ml chloroform:methanol (1:1, v/v) followed by 1 ml of methanol. The combined eluates were dried in vacuo, and the ceramide mass was quantified by long chain bases released after an acid hydrolysis in 0.5 M HCl in methanol at 65°C for 15 h [40]. Free long chain bases were analyzed as described by Lauter & Trams [41]. The contents of phospholipids in chromatographic fractions were determined by the method of [42]. Samples for isolation of free [^14^C[sphingosine (Sph) were prepared as recommended in [43] and Sph separation was performed by TLC in the solvent system: chloroform/methanol/ammonium hydroxide (40:10:1, v/v). The appropriate standards were applied on each plate for quantification. The gel spots containing [^14^C]lipids were scraped and transferred to scintillation vials. Radioactivity was measured by a scintillation counter.

### Statistical analyses

One-way analysis of variance (ANOVA) procedures was used to assess significant differences among treatment groups. For each significant effect of treatment, the Fisher LSD and Turky HSD tests were used for comparison of multiple group means. Student's *t-*test was used for paired observations and significance was set at *p *< 0.05 and *p *< 0.001.

## Authors' contributions

NAB conceived the study and participated in its design, coordination, and the manuscript preparation. EGS participated in the data collection and performed the statistical analysis.
